# Gotong royong and COVID-19 vaccination in Indonesia: Linking communal values to collective immunity

**DOI:** 10.1016/j.puhip.2025.100677

**Published:** 2025-10-30

**Authors:** Abram L. Wagner, Marie Jacobson, Renie Cuyno Mellen, Widia Adiratna, Emily Treleaven, Aditya L. Ramadona, Retna Siwi Padmawati, Yayi Suryo Prabandari

**Affiliations:** a Department of Epidemiology, University of Michigan, Ann Arbor, USA; b Central of Health Behaviour and Promotion, Faculty of Medicine, Public Health and Nursing, Universitas Gadjah Mada, Yogyakarta, Indonesia; c Department of Health Behaviour, Environment and Social Medicine, Faculty of Medicine, Public Health and Nursing, Universitas Gadjah Mada, Yogyakarta, Indonesia; d Institute for Social Research, University of Michigan, Ann Arbor, USA; e Universitas Islam Negeri Walisongo, Semarang, Indonesia

**Keywords:** COVID-19 vaccines, Community engagement, Vaccine, Indonesia, Theory of planned behavior

## Abstract

**Objective:**

This study examines the relationship between gotong royong participation and COVID-19 vaccine uptake in Yogyakarta, Indonesia.

**Study design:**

Cross-sectional survey.

**Methods:**

We conducted a cross-sectional survey of 804 adults across rural and urban subdistricts in Yogyakarta province. The survey assessed COVID-19 vaccination status, engagement in gotong royong activities, and demographic, experiential, and normative factors related to vaccination. Multivariable logistic regression models were used to identify associations between gotong royong involvement and vaccination outcomes.

**Results:**

Participation in *gotong royong* was reported by 44.3 % of respondents, with the most common activity being support and assistance (39.2 %). Among all participants, 10.8 % were unvaccinated, 43.9 % completed the primary series, and 41.8 % had received a booster dose. Individuals involved in gotong royong were less likely to be unvaccinated (5.3 % vs. 15.2 %) and, in multivariable analysis, had significantly higher odds of receiving at least one vaccine dose (OR: 3.41; 95 % CI: 1.78–6.54). They were also more likely to report community encouragement for vaccination, including from religious leaders (55.6 % vs. 40.2 %, P < 0.0001) and peers (83.7 % vs. 77.0 %, P = 0.0027).

**Conclusions:**

These findings highlight the importance of aligning vaccination campaigns with culturally embedded social structures. Leveraging communal values and trusted local actors may enhance vaccine uptake in collectivist settings and inform broader strategies for routine immunization and pandemic preparedness.

**What this study adds**

• Identifies a strong, positive association between participation in gotong royong, a traditional Indonesian form of communal cooperation, and COVID-19 vaccine uptake.
• Provides empirical evidence that collectivist cultural norms and community-based engagement are important predictors of health behaviour in non-Western settings.
• Demonstrates that pro-vaccine social norms and encouragement from local religious and community leaders are closely tied to communal practices and vary by geographic context.

**Implications for Policy and Practice -**

• Vaccination campaigns in collectivist societies may be more effective when they are embedded within existing social structures and leverage trusted community figures.
• Public health strategies that promote vaccination should align with cultural values of mutual aid and shared responsibility, especially in under-vaccinated or rural areas.
• Tailored, place-sensitive interventions could support uptake of future vaccines, including boosters and routine immunizations.

## 1 Introduction

The COVID-19 pandemic has strained global health systems, with vaccination reducing transmission and severe illness [1] In Indonesia, early mandates led to widespread initial uptake, but voluntary booster doses have seen reduced participation, particularly in rural areas [2,3]. Barriers such as misinformation, logistical issues, and distrust contribute to persistent vaccine hesitancy despite strong community ties.

While Western models like the Theory of Planned Behaviour (TPB) and Health Belief Model (HBM) have dominated vaccine hesitancy research [4,5], they focus on individual attitudes and risk assessments. In collectivist societies, however, health decisions are deeply embedded in social relationships and communal obligations. Frameworks such as social capital theory [6,7], collective action theory [8], and the moral economy of health [9] offer more appropriate lenses for understanding vaccine behaviour in Indonesia, where decisions are shaped by trust, reciprocity, and ethical responsibility to the group.

The Indonesian cultural practice of *gotong royong* emphasizes communal cooperation, mutual aid, and reciprocity. Rooted in a sense of collective responsibility, *gotong royong* plays a central role in Indonesian society by fostering solidarity and shared obligations within communities [10]. Traditionally, it has been applied to communal labor projects, disaster recovery efforts, and informal social support networks [11,12]. As a form of collective action, *gotong royong* can enhance community resilience, especially in rural areas where formal health infrastructure may be limited but social ties are strong [13]. It has also been shown to increase enthusiasm for public works and health initiatives that offer shared community benefit [14,15].

Given its deep roots in communal responsibility, *gotong royong* provides a culturally grounded framework for understanding vaccine acceptance and hesitancy in Indonesia. With vaccination conferring benefits beyond the individual, this tradition of mutual aid has been increasingly recognized as a valuable mechanism for mobilizing collective action in public health. Recognizing the power of *gotong royong*, the Indonesian government leveraged it as a policy tool to expand vaccine access. The Vaksinasi Gotong Royong (VGR) program enabled private businesses to provide company-funded vaccinations for employees, their families, and surrounding communities, aiming to accelerate herd immunity [16]. At the community level, *gotong royong* has also been mobilized to facilitate vaccination efforts, including organizing transportation to vaccine sites, assisting with registration, spreading vaccine-related information, and supporting COVID-19 contact tracing and isolation efforts [17].

Beyond logistical support, *gotong royong* may shape health behaviours and vaccine decision-making by embedding these choices within social expectations and collective norms. Studies suggest that social capital gained through community participation plays a crucial role in health outcomes. For example, mothers with higher social capital are more likely to seek healthcare for their children [18], and engagement in community organizations has been linked to improved child health status, even among families with limited financial resources [19]. Similarly, social trust (closely tied to *gotong royong*) has been associated with better mental health outcomes and stronger adherence to public health measures [20].

Western behavioural models often fail to capture these relational dynamics. While TPB has been applied to COVID-19 vaccination [[21], [22], [23]], it underemphasizes the role of collective norms. Few studies incorporate community-driven cultural frameworks [24,25], despite evidence that subjective norms may outweigh individual attitudes in collectivist settings [26].

This study examines how participation in gotong royong is associated with COVID-19 vaccine uptake in Yogyakarta province. We argue that communal engagement fosters social trust, reinforces prosocial norms, and positions vaccination as a collective responsibility. By integrating social capital and moral economy frameworks, this study advances an alternative, culturally grounded understanding of vaccine decision-making.

## 2 Methods

### 2.1 Study population

This quantitative study utilized multistage sampling to select the study sample from the Bantul and Gunungkidul districts in Yogyakarta province, Indonesia. These districts were chosen because they had the lowest COVID-19 vaccination coverage [27]. In February 2022, prior to data collection, Bantul had 65 % first dose and 50 % second dose coverage among adults aged 19–60 years, while Gunungkidul had 55 % first dose and 35 % second dose coverage in this age group [27]. Moreover, in 2021, Gunungkidul exhibited relatively low vaccine acceptance rates (MOH Republic Indonesia, 2022), particularly in rural areas [2,3].

### 2.2 Sampling and data collection

In collaboration with local authorities, we purposefully selected 16 sub-districts (8 rural, 8 urban) based on social determinants and intervention feasibility. Within these, village office population data from 4 areas was randomized to generate a list of potential respondents. The final respondent list incorporated sub-village head data and categorization. Screening preceded the survey to confirm household categorization. Households were systematically randomly sampled from this list, with up to 5 visits to initially non-responsive households to reduce non-response.

Assuming 70 % national booster coverage [27], we calculated a required 646 household sample for a 5 % margin of error with 80 % power and an alpha of 0.05. Our target final sample size was slightly increased to 800. We aimed to purposively select diverse households (half rural, half urban), targeting households with spouses, multigenerational families, and young adults cohabiting with parents over 40.

To develop our survey instrument, we conducted a systematic literature review on vaccine hesitancy enablers and barriers in lower-middle income countries. At the time of the study, we did not identify existing validated instruments specifically examining *gotong royong* in relation to vaccination. As an early empirical study on this concept, we developed a tailored questionnaire focused on the Indonesian context. In-depth interviews with local residents, stakeholders (Health Office, Puskesmas, NGOs, PKBI, village heads, community leaders, religious leaders, school teachers, cultural leaders, youth leaders) informed understanding of issues in Gunungkidul. We did not conduct formal psychometric testing, but these individuals were consulted on face validity of the questionnaire.

Over 6 months, we conducted 800 survey interviews with household members, either online or in-person, with the data collection mode based on levels of COVID-19 in the community. Enumerators underwent day-long training by the study team on understanding questions, answer options, usage of the data collection tool, potential respondent answers, and practice interviews. Established protocols included protective equipment and testing staff for COVID-19.

### 2.3 Measures

The primary outcome was whether participants had received at least one dose of a COVID-19 vaccine, based on self-report. Vaccines commonly used in Indonesia include AstraZeneca, Moderna, Sinopharm, Pfizer, and Sinovac. Participants were asked how many doses they had received. Based on responses, we also constructed a categorical variable with four groups: unvaccinated, partially vaccinated (1 dose), completed primary series (2 doses), and boosted (≥1 additional dose after the primary series).

The key independent variable was participation in *gotong royong*, Indonesia's traditional communal cooperation system. Participants were asked if they were involved in any communal activity in the following areas: pandemic support & assistance, vaccine information & registration, communication & advocacy, COVID-19 contact tracing & response, and support for isolation & healthcare. A binary indicator was created for involvement in any *gotong royong* activity.

We also assessed COVID-19-related experiences and beliefs. Participants reported prior infection with COVID-19. Perceived social norms were measured through questions on encouragement from religious and community leaders, friends and neighbours, and health workers (categorized as positive, somewhat positive, or somewhat negative).

Community engagement was measured by whether participants had received COVID-19 education from local leaders. For vaccinated individuals, we captured who recommended or provided advice about vaccination (health workers, religious leaders, community leaders, or others). Intentions to vaccinate were assessed using hypothetical scenarios involving improved access or more scientific information.

Demographic and socioeconomic variables included gender; age (categorized as 18–29, 30–39, 40–49, 50–59, 60–69, and 70+); and education level (from no schooling to higher education). Religion was coded as Muslim vs. non-Muslim; marital status as married, separated/divorced, widowed, or never married. Occupation was grouped into broad categories (e.g., agriculture, business/sales, education, domestic work, unemployed). Household income was categorized relative to the Indonesian poverty line (2,050,000 IDR; ∼$128.27 USD) (BPS, 2023). We also included leadership role variables (e.g., youth, women's, religious, health) and constructed indicators for reasons for vaccination and roles in *gotong royong*-related support. Urbanicity was coded as urban vs. rural based on location of interview.

### 2.4 Statistical analysis

Descriptive statistical analyses were conducted to examine the characteristics of the study population. Bivariate analyses, including chi-square tests, were employed to assess relationships between sociodemographic factors, *gotong royong* involvement, and COVID-19 vaccination outcomes.

Multivariable logistic regression analyses were performed to investigate the association between involvement in *gotong royong* activities and COVID-19 vaccination while controlling for relevant covariates. The covariates included in the model were urbanicity, occupation, age, education, gender, religion, income, marital status, chronic disease history, community role, and leadership role.

We analysed participants’ stated reasons for receiving a COVID-19 vaccine, comparing groups by *gotong royong* involvement and urbanicity to explore how community participation and location influenced vaccination motivations. Chi-square tests were used to assess statistical significance.

Throughout the statistical analyses, two-sided p-values less than 0.05 were considered statistically significant. Analyses were conducted using SAS software.

## 3 Results

In total, 808 individuals were approached to participate in the study, and 804 (99.5 %) agreed. Table 1 describes respondents’ socio-demographic characteristics. Participants were evenly distributed by urbanicity (50.1 % urban, 49.9 % rural) and gender (49.9 % men and 50.1 % women).

**Table 1:** Demographic distribution of study population, Gunungkidul, Yogyakarta, Indonesia, 2023 (N = 804).

Demographic variable	Category	Count (%)
Urbanicity	Urban	403 (50.1)
	Rural	401 (49.9)
Occupation	Education, Healthcare, and Public Service	51 (6.3)
	Agriculture and Labor	203 (25.3)
	Homemakers	215 (26.7)
	Business and Private Sector	232 (28.9)
	Retired and unemployed	63 (7.8)
	Other	40 (5.0)
Age	18–29 years	89 (11.1)
	30–39 years	169 (21.0)
	40–49 years	239 (29.7)
	50–59 years	180 (22.4)
	60–69 years	100 (12.4)
	≥70 years	27 (3.4)
Education	Did not finish elementary school	38 (4.73)
	Finished elementary school/equivalent	220 (27.36)
	Finished junior high school/equivalent	172 (21.39)
	Graduated high school/equivalent	280 (34.83)
	Diploma/Higher education	94 (11.69)
Gender	Male	401 (49.9)
	Female	403 (50.1)
Religion	Muslim	792 (98.5)
	Not Muslim	12 (1.5)
Income	<2,050,000 Rupiah	595 (74.0)
	≥2,050,001 Rupiah	209 (26.0)
Marital status	Not married yet	58 (7.2)
	Divorced	33 (4.1)
	Married	713 (88.7)
History of chronic disease	No	705 (87.7)
	Yes	99 (12.3)
Leadership role in community	Youth leaders	56 (7.0)
	Community Leaders	82 (10.2)
	Women Leaders	37 (4.6)
	Religious, Cultural figures	12 (1.5)
	Health figures	33 (4.1)
	No role in community	601 (74.8)
	Other	6 (0.8)
Leadership roles	Any	168 (20.9)
	None	636 (79.1)

In total, 44.3 % of participants were active in any *gotong royong* activity. The most common types included support and assistance (39.2 %), vaccination information and registration (16.5 %), and communication and advocacy (16.4 %). Urban communities had higher participation rates than rural areas, with 47.9 % of urban residents involved compared to 40.7 % in rural communities. Urban participants were more likely to engage in support and advocacy roles, while rural participants were more involved in vaccine registration. Additionally, men were more likely to participate than women, with 59 % of males engaged in *gotong royong* compared to 29 % of females (Table 2).

**Table 2:** Relationship between demographic variables and *Gotong Royong* involvement, Gunungkidul, Yogyakarta, Indonesia, 2023 (N = 804).

Demographic Variable	Category	Type of *Gotong Royong* activity [Count (%)]					Any type of *Gotong Royong*	
		Support & Assistance	Vaccine info & registration	Communication & Advocacy	COVID-19 Tracing & Response	Support for Isolation & Healthcare	Count (%)	P-value
Overall		315 (39.2)	133 (16.5)	132 (16.4)	29 (3.6)	42 (5.2)	356 (44.3)	
Urbanicity	Urban	185 (45.9)	48 (11.9)	81 (20.1)	16 (4.0)	23 (5.7)	193 (47.9)	0.0387
	Rural	130 (32.4)	85 (21.2)	51 (12.7)	13 (3.2)	19 (4.7)	163 (40.7)	
Occupation	Education, Healthcare, and Public Service	29 (56.9)	18 (35.3)	16 (31.4)	2 (3.9)	6 (11.8)	32 (62.8)	<.0001^MC^
	Agriculture and Labor	79 (38.9)	20 (9.9)	28 (13.8)	7 (3.5)	7 (3.5)	84 (41.4)	
	Homemakers	54 (25.1)	29 (13.5)	30 (14.0)	3 (1.4)	7 (3.3)	64 (29.8)	
	Business and Private Sector	104 (44.8)	46 (19.8)	38 (16.4)	14 (6.0)	15 (6.5)	125 (53.9)	
	Retired and unemployed	25 (39.7)	10 (15.9)	9 (14.3)	0 (0.0)	2 (3.2)	26 (41.3)	
	Other	24 (60.0)	10 (25)	11 (27.5)	3 (7.5)	5 (12.5)	25 (62.5)	
Age	18–29 years	39 (43.8)	13 (14.6)	13 (14.6)	1 (1.1)	1 (1.1)	45 (50.6)	<.0001^MC^
	30–39 years	78 (46.2)	32 (18.9)	30 (17.8)	4 (2.4)	7 (4.1)	86 (50.9)	
	40–49 years	112 (46.2)	50 (20.9)	48 (20.1)	16 (6.7)	17 (7.1)	126 (52.7)	
	50–59 years	58 (46.9)	28 (15.6)	28 (15.6)	6 (3.3)	15 (8.3)	68 (37.8)	
	60–69 years	26 (26.0)	10 (10.0)	12 (12.0)	1 (1.0)	1 (1.0)	28 (28.0)	
	≥70 years	2 (7.4)	0 (0.0)	1 (3.7)	1 (3.7)	1 (3.7)	3 (11.1)	
Education	Did not finish elementary school	5 (13.16)	2 (5.26)	2 (5.26)	1 (2.63)	1 (2.63)	5 (13.16)	<.0001^MC^
	Finished elementary school/equivalent	44 (20.00)	15 (6.82)	19 (8.64)	7 (3.18)	4 (1.82)	53 (24.09)	
	Finished junior high school/equivalent	60 (34.88)	30 (17.44)	27 (15.70)	6 (3.49)	7 (4.07)	74 (43.02)	
	Graduated high school/equivalent	156 (55.71)	57 (20.36)	61 (21.79)	13 (4.64)	22 (7.86)	171 (61.07)	
	Diploma/Higher education	50 (53.19)	29 (30.85)	23 (24.47)	2 (2.13)	8 (8.51)	53 (56.38)	
Gender	Male	225 (56.1)	68 (17.0)	81 (20.2)	26 (6.5)	32 (8.0)	238 (59.4)	<.0001^MC^
	Female	90 (22.3)	65 (16.1)	51 (12.7)	3 (0.7)	10 (2.5)	118 (29.3)	
Religion	Muslim	309 (39.0)	131 (16.5)	130 (16.4)	29 (3.7)	41 (5.2)	350 (44.2)	0.7736^MC^
	Not Muslim	6 (50.0)	2 (16.7)	2 (16.7)	0 (0.0)	1 (8.3)	6 (50.0)	
Income	<2,050,000 Rupiah	198 (33.3)	83 (14.0)	82 (13.8)	19 (3.2)	24 (4.0)	233 (39.2)	<0.0001
	≥2,050,001 Rupiah	117 (56.0)	50 (23.9)	50 (23.9)	10 (4.8)	18 (8.6)	123 (58.9)	
Marital status	Not married yet	28 (48.3)	7 (12.1)	7 (12.1)	0 (0.0)	1 (1.7)	31 (53.5)	0.0007^MC^
	Divorced	5 (15.2)	1 (3.0)	2 (6.1)	0 (0.0)	0 (0.0)	5 (15.2)	
	Married	282 (39.6)	125 (17.5)	123 (17.3)	29 (4.1)	41 (5.8)	320 (44.9)	
History of chronic disease	No	294 (41.7)	124 (17.6)	122 (17.3)	24 (3.4)	40 (5.7)	334 (47.4)	<.0001^MC^
	Yes	21 (21.2)	9 (9.1)	10 (10.1)	5 (5.1)	2 (2.0)	22 (22.2)	
Leadership role in community	Youth Leaders	39 (69.6)	14 (25.0)	13 (23.2)	5 (8.9)	5 (8.9)	42 (75.0)	<.0001^MC^
	Community Leaders	65 (79.3)	32 (39.0)	34 (41.5)	7 (8.5)	14 (17.1)	69 (84.2)	<.0001^MC^
	Women Leaders	14 (37.8)	8 (21.6)	7 (18.9)	0 (0.0)	2 (5.4)	15 (40.5)	<.7356^MC^
	Religious/Cultural figures	10 (83.3)	6 (50.0)	6 (50.0)	3 (25.0)	4 (33.3)	10 (83.3)	<.0073^MC^
	Health figures	17 (51.5)	19 (57.6)	22 (66.7)	0 (0.0)	2 (6.1)	27 (81.8)	<.0001^MC^
	No role	182 (30.3)	63 (10.5)	61 (10.2)	15 (2.5)	20 (3.3 %)	205 (34.1)	<0.0001
	Other	4 (66.7)	1 (16.7)	2 (33.3)	1 (16.7)	1 (16.7)	5 (83.3)	.0932^MC^
Leadership role	Any	113 (67.3)	50 (29.8)	50 (29.8)	11 (6.6)	18 (10.7)	121 (72.0)	<0.0001
	None	202 (31.8)	83 (13.1)	82 (12.9)	18 (2.8)	24 (3.8)	235 (37.0)	

Note: ^MC^=Monte Carlo estimation of exact p-value’

Caption: The most common type of gotong royong activity was support and assistance tasks. Participation in any gotong royong activity was higher in urban than rural areas.

Table 3 shows that participation in *gotong royong* was significantly associated with COVID-19 vaccination. Those involved had over three times the odds of being vaccinated compared to those not involved (OR = 3.41; 95 % CI: 1.78, 6.54). Higher education was linked to lower uptake; individuals with a diploma or more had lower odds than high school graduates (OR = 0.13; 95 % CI: 0.04, 0.38). Men were less likely to be vaccinated than women (OR = 0.28; 95 % CI: 0.14, 0.58), and individuals with chronic conditions also had lower odds of vaccination (OR = 0.21; 95 % CI: 0.11, 0.40). Urbanicity was not significantly associated with vaccine uptake (OR = 1.33; 95 % CI: 0.77, 2.30).

**Table 3:** Relationship between demographic variables, *gotong royong* involvement and COVID-19 vaccination, Gunungkidul, Yogyakarta, Indonesia, 2023 (N = 804).

Demographic Variable	Category	Received at least one dose	
		OR	UL, LL
*Gotong Royong*	Not involved	reference	
	Involved	3.41	1.78, 6.54
Urbanicity	Urban	1.33	0.77, 2.30
	Rural	reference	
Occupation	Education, Healthcare, and Public Service	reference	
	Agriculture and Labor	1.42	0.70, 2.87
	Homemakers	1.40	0.59, 3.31
	Business and Private Sector	3.02	0.86, 10.68
	Retired and unemployed	1.92	0.41, 8.90
Age	Other	1.57	0.27, 9.16
	18–29 years	1.14	0.51, 2.53
	30–39 years	reference	
	40–49 years	1.07	0.53, 2.12
	50–59 years	0.83	0.36, 1.92
	60–69 years	3.06	0.70, 13.30
Education	≥70 years	1.16	0.38, 3.50
	Did not finish elementary school	0.47	0.20, 1.07
	Finished elementary school/equivalent	0.85	0.36, 1.97
	Finished junior high school/equivalent	reference	
	Graduated high school/equivalent	0.13	0.04, 0.38
Gender	Male	0.28	0.14, 0.58
	Female	reference	
Income	<2,050,000 Rupiah	reference	
	≥2,050,001 Rupiah	0.72	0.36, 1.45
Marital status	Not married yet	0.47	0.07, 2.96
	Divorced	2.66	0.69, 10.30
	Married	reference	
History of chronic disease	No	reference	
	Yes	0.211	0.11, 0.40
Leadership role	Any	1.31	0.63, 2.71
	None	reference	

Caption: Involvement in any type of gotong royong activity was associated with greater odds of having received any dose of the COVID-19 vaccine.

Overall, 41.8 % of individuals had a booster dose, 43.9 % completed primary series (without a booster dose) and 10.8 % were totally unvaccinated (Table 4). Participation in *gotong royong* was associated with higher rates of completing the primary series, though it was less strongly linked to booster uptake (P < 0.0001). Urban residents were more likely to receive a booster dose than rural residents (47.6 % vs. 35.9 %), and less likely to remain unvaccinated (7.7 % vs. 14.0 %). Booster uptake was also high (90 %) among those working in academia, healthcare, government, and public service.

**Table 4:** Demographic variables, *Gotong Royong*, and Vaccination Status, Gunungkidul, Yogyakarta, Indonesia, 2023.

		Unvaccinated	Partially vaccinated	Completed primary series	Has 1 or 2 booster doses	P-value
Overall		87 (10.8 %)	28 (3.5 %)	353 (43.9 %)	336 (41.8 %)	
Involved in any *Gotong Royong* activity	Yes	19 (5.3 %)	15 (4.2 %)	173 (48.6 %)	149 (41.9 %)	<0.0001
	No	68 (15.2 %)	13 (2.9 %)	180 (40.2 %)	187 (41.7 %)	
Urbanicity	Urban	31 (7.7 %)	16 (4.0 %)	164 (40.7 %)	192 (47.6 %)	0.0009
	Rural	56 (14.0 %)	12 (3.0 %)	189 (47.1 %)	144 (35.9 %)	
Occupation	Education, Healthcare, and Public Service	0 (0.0 %)	1 (2.0 %)	4 (7.8 %)	46 (90.2 %)	<.0001^MC^
	Agriculture and Labor	33 (16.3 %)	7 (3.5 %)	109 (53.7 %)	54 (26.6 %)	
	Homemakers	19 (8.8 %)	9 (4.2 %)	92 (42.8 %)	95 (44.2 %)	
	Business and Private Sector	29 (12.5 %)	8 (3.5 %)	108 (46.6 %)	87 (37.5 %)	
	Retired and unemployed	4 (6.4 %)	1 (1.6 %)	25 (39.7 %)	33 (52.4 %)	
	Other	2 (5.0 %)	2 (5.0 %)	15 (37.5 %)	21 (52.5 %)	
Age	18–29 years	4 (4.5 %)	4 (4.5 %)	49 (55.1 %)	32 (36.0 %)	0.0051^MC^
	30–39 years	11 (6.5 %)	6 (3.6 %)	74 (43.8 %)	78 (46.2 %)	
	40–49 years	22 (9.2 %)	10 (4.2 %)	109 (45.6 %)	98 (41.0 %)	
	50–59 years	24 (13.3 %)	4 (2.2 %)	79 (43.9 %)	73 (40.6 %)	
	60–69 years	22 (22.0 %)	2 (2.0 %)	36 (36.0 %)	40 (40.0 %)	
	≥70 years	4 (14.8 %)	2 (7.4 %)	6 (22.2 %)	15 (55.6 %)	
Education	Did not finish elementary school	15 (39.47)	3 (7.89)	88 (51.16)	63 (36.63)	<.0001^MC^
	Finished elementary school/equivalent	36 (16.36)	5 (2.27)	99 (45.00)	80 (36.36)	
	Finished junior high school/equivalent	14 (8.14)	7 (4.07)	88 (51.16)	63 (36.63)	
	Graduated high school/equivalent	17 (6.07)	12 (4.29)	143 (51.07)	108 (38.57)	
	Diploma/Higher education	5 (5.32)	1 (1.06)	16 (17.02)	72 (76.60)	
Gender	Male	55 (13.7 %)	15 (3.7 %)	197 (49.1 %)	134 (33.4 %)	<0.0001
	Female	32 (7.9 %)	13 (3.2 %)	156 (38.7 %)	202 (50.1 %)	
Religion	Muslim	87 (11.0 %)	28 (3.5 %)	346 (43.7 %)	331 (41.8 %)	0.6731^MC^
	Not Muslim	0 (0.0 %)	0 (0.0 %)	7 (58.3 %)	5 (41.7 %)	
Income	<2,050,000 Rupiah	66 (11.1 %)	23 (3.9 %)	276 (46.4 %)	230 (38.7 %)	0.0212
	≥2,050,001 Rupiah	21 (10.1 %)	5 (2.4 %)	77 (36.8 %)	106 (50.7 %)	
Marital status	Not married yet	4 (6.9 %)	2 (3.5 %)	32 (55.2 %)	20 (34.5 %)	0.1145^MC^
	Divorced	3 (9.1 %)	3 (9.1 %)	9 (27.3 %)	18 (54.6 %)	
	Married	80 (11.2 %)	23 (3.2 %)	312 (43.8 %)	298 (41.8 %)	
History of chronic disease	No	58 (8.2 %)	24 (3.4 %)	320 (45.4 %)	303 (43.0 %)	<.0001^MC^
	Yes	29 (29.3 %)	4 (4.0 %)	33 (33.3 %)	33 (33.3 %)	
Role in community	Youth Leaders	3 (5.4 %)	2 (3.6 %)	28 (50.0 %)	23 (41.1 %)	0.5262^MC^
	Community Leaders	7 (8.5 %)	2 (2.4 %)	38 (46.3 %)	35 (42.7 %)	0.8953^MC^
	Women Leaders	3 (8.1 %)	0 (0.0 %)	14 (37.8 %)	20 (54.1 %)	0.4664^MC^
	Religious/Cultural figures	1 (8.3 %)	0 (0.0 %)	5 (41.7 %)	6 (50.0 %)	0.9450^MC^
	Health figures	1 (3.0 %)	0 (0.0 %)	14 (42.4 %)	18 (54.6 %)	0.2892^MC^
	No role	72 (12.0 %)	23 (3.8 %)	264 (43.9 %)	242 (40.3 %)	0.1653
	Other	0 (0.0 %)	0 (0.0 %)	3 (50.0 %)	3 (50.0 %)	1.0000^MC^

Note: ^MC^=Monte Carlo estimation of exact p-value.

Caption: Involvement in gotong royong was more strongly related to receipt of the primary COVID-19 vaccination series than booster doses.

Table 5 highlights how *gotong royong* involvement relates to vaccination motivations. Participants active in *gotong royong* were less likely to have had COVID-19 and more likely to report encouragement from religious and community leaders, health workers, and peers. They were also more likely to say they would vaccinate if given more scientific information.

**Table 5:** Description of reasons to obtain a COVID-19 vaccine, by *Gotong Royong* involvement and urbanicity, Gunungkidul, Yogyakarta, Indonesia, 2023.

	Any *Gotong Royong* involvement Count (column %)			Urbanicity Count (column %)		
	Yes (n = 356)	No (n = 448)	P-value	Urban (N = 403)	Rural (N = 401)	P-value
History of contracting COVID-19	39 (11.0)	84 (18.8)	0.0005	74 (18.4)	49 (12.2)	0.0524
Religious leaders in the community want you to be vaccinated	198 (55.6)	180 (40.2)	<0.0001	318 (78.9)	60 (15.0)	<0.0001
Community leaders want you to be vaccinated	337 (94.7)	414 (92.4)	0.2011	366 (90.8)	385 (96.0)	0.0030
Friends, colleagues and neighbours you know would want to be vaccinated if recommended a vaccine	298 (83.7)	345 (77.0)	0.0027	271 (67.3)	372 (92.8)	<0.0001
Health workers in your community want you to be vaccinated	301 (84.6)	323 (72.1)	<0.0001	345 (85.6)	279 (69.6)	<0.0001
Received education from the local community	332 (93.3)	400 (89.3)	0.0500	361 (89.6)	371 (92.5)	0.1443
You will be vaccinated if someone delivers it to the vaccine site	188 (52.8)	238 (53.1)	0.9289	217 (53.9)	209 (52.1)	0.6238
You will be vaccinated if you get more scientific and medical information about vaccines	238 (66.9)	255 (56.9)	0.0041	255 (63.3)	238 (59.4)	0.2534
You vaccinate because it is recommended by health workers	42 (11.8)	44 (9.8)	0.3678	63 (15.6)	23 (5.7)	<0.0001
You vaccinate according to the advice of religious and community leaders	35 (9.8)	51 (11.4)	0.4793	57 (14.1)	29 (7.2)	0.0015

Caption: Involvement in gotong royong activity was also associated with other pro-social vaccination attitudes and experiences, like stating religious leaders or health workers in your community want you to be vaccinated. Compared to those in urban areas, people in rural areas were less likely to say that religious leaders wanted them vaccinated, but more likely to say that community leaders wanted them to be vaccinated.

Urban-rural differences were also pronounced. Urban residents more frequently cited support from religious leaders (78.9 % vs. 15.0 %), health worker recommendations (15.6 % vs. 5.7 %), and advice from local leaders (14.1 % vs. 7.2 %) as reasons for vaccination. Rural participants more often perceived that their friends and neighbours supported vaccination (92.8 % vs. 67.3 %) and reported encouragement from community health workers (85.6 % urban vs. 69.6 % rural).

## 4 Discussion

This study identified a significant positive association between participation in *gotong royong*, Indonesia's traditional practice of mutual aid, and COVID-19 vaccine uptake in Yogyakarta. Individuals involved in *gotong royong* were more receptive to vaccination, influenced by social norms and trusted messengers such as religious leaders and health workers. These findings highlight that in collectivist societies, health decisions are shaped by social expectations and shared obligations, and not just individual attitudes. Public health campaigns that emphasize community protection, rather than personal risk, may be more effective in such contexts.

While most health behaviour research draws from Western, individualist models, our findings underscore the importance of collectivist frameworks [28,29]. Prior work has shown that collectivist values, like concern for others and social harmony, are linked to higher vaccine acceptance. This study contributes to that growing body of literature by showing how *gotong royong*, a form of collective action, can be a critical driver of vaccination in Indonesia.

### 4.1 The role of social capital and collectivist norms in vaccine uptake

Individuals engaged in *gotong royong* activities exhibited greater receptiveness to vaccination, influenced by recommendations from religious and community leaders, perceived social norms favouring vaccination, and guidance from health workers. Our findings align more closely with social capital theory [6,7] and collective action models [8] than with traditional individual-level behavioural theories like the Theory of Planned Behaviour [30]. In Indonesia's interdependent social context, community trust and group identity significantly influence health behaviours. Endorsements from religious or community leaders, as well as perceptions of social norms, were closely tied to vaccine uptake.

The Global Flourishing Survey [31] affirms that religious beliefs and interpersonal trust shape health behaviour globally. In contexts like Indonesia, where religion and community participation are central to social life, these relational factors may outweigh individual risk assessments in vaccination decisions. A study of COVID-19 vaccination in Hong Kong found that vaccine acceptors had high agreement with measures of vaccination as a collective good [32]. Our results suggest that health promotion strategies in LMICs may be more successful when they build on existing social institutions and norms.

### 4.2 Demographic factors and vaccine uptake

The contrasting patterns observed between urban and rural areas highlight the different ways social influence and leadership function in shaping vaccination motivation, reinforcing the need for localized public health strategies. While rural communities exhibited lower vaccination rates, they also displayed stronger normative beliefs about vaccination, with individuals more likely to perceive that their friends, colleagues, and neighbours would want to be vaccinated if recommended a vaccine. In contrast, urban vaccination decisions appeared to be more strongly influenced by religious leaders, underscoring how authority figures play a distinct role in shaping vaccine confidence in urban settings. This aligns with findings from another study during the COVID-19 pandemic in Indonesia, who documented higher levels of social solidarity in rural compared to urban areas [13]. These differences in social cohesion and trust dynamics underscore the importance of designing vaccination efforts that resonate with the specific social fabric of each context: peer-driven approaches may be more effective in rural areas, while institutional messaging and endorsements from religious figures may carry more weight in urban communities.

Other demographic characteristics were associated with vaccination uptake. Men less likely to be vaccinated than women, possibly reflecting that risk perceptions and social norms vary by gender [33]. Similarly, individuals in education, healthcare, and public service roles had higher uptake, likely due to increased exposure to health information and institutional mandates [34]. Those with lower education levels were less likely to be vaccinated, pointing to persistent challenges with health literacy and trust in formal institutions [35]. By income, higher-income individuals had greater vaccination rates. In low-income populations, barriers such as transportation costs, lost wages, and limited health information could hinder uptake [33]. Older adults were more likely to be vaccinated, possibly due to targeted outreach and higher risk of severe disease, but were less likely to participate in gotong royong activities, suggesting a potential opportunity to use communal engagement strategies to reach younger adults more effectively.

These differences in vaccine uptake suggest that vaccination efforts must be tailored to the unique social and geographic context, leveraging peer-driven norms in rural areas and institutional messaging in urban areas, while also addressing structural barriers through logistical support (e.g., mobile clinics, workplace vaccination) and targeted information campaigns.

### 4.3 Leveraging gotong royong for vaccine promotion

Despite high engagement in gotong royong, its potential has not been fully realized in promoting vaccination. While gotong royong has historically been applied to disaster response and recovery efforts, including during the acute phases of the COVID-19 pandemic [13], there is limited prior research examining its application to vaccination specifically. A pandemic setting may lend itself more naturally to mutual aid efforts than routine immunization programs, where perceived risk is lower and social mobilization less intense [36]. As such, different forms of vaccine hesitancy may emerge in routine settings, and gotong royong may not be as readily leveraged without deliberate planning. To strengthen this link, public health strategies could integrate *gotong royong* into community-based outreach, using it not only for logistics but also for trust-building and norm-setting. Potential approaches include partnering with trusted local leaders as vaccine advocates, embedding education programs within existing communal structures, and disseminating culturally resonant messages through social networks. Drawing from successful community engagement models [37], this integration could help normalize vaccination as a shared responsibility and enhance uptake, particularly in rural or under-vaccinated communities. Moreover, expanding gotong royong-informed strategies to other health domains, such as routine immunization and maternal care, may contribute to long-term resilience in Indonesia's public health system.

### 4.4 Rural and urban vaccine outreach strategies

The contrasting patterns observed between urban and rural communities reinforce the need for localized public health strategies that align with distinct social structures and leadership influences. While rural areas reported lower vaccination rates, they also exhibited stronger communal ties and peer-driven normative beliefs about vaccination, suggesting that social cohesion is an underutilized resource in vaccine promotion. In contrast, urban communities were more reliant on top-down messaging from religious and institutional leaders, highlighting the importance of authority figures in shaping vaccine confidence in these settings.

These findings suggest that rural vaccination efforts should emphasize community-based engagement, where trusted local figures, such as village leaders, religious authorities, and grassroots organizations, can help disseminate accurate vaccine information through horizontal social networks. By amplifying local vaccine uptake norms and reinforcing community expectations around immunization, rural outreach efforts can better align with existing social structures. Meanwhile, urban vaccination campaigns may be more effective when strengthening public trust through endorsements from religious and institutional leaders, as these figures appear to play a more central role in shaping vaccine attitudes.

### 4.5 Strengths and limitations

This study's strengths include a high response rate (98 %), and enrolment of members from the community based on address-based sampling.

However, limitations include the regional focus on Yogyakarta, which may not generalize across Indonesia's diverse settings. The use of self-reported data may introduce bias, and the cross-sectional design limits causal interpretation. We also did not have information on the intensity or frequency of *gotong royong* involvement.

Future research should explore longitudinal trends in vaccine attitudes and assess how *gotong royong* can be more effectively integrated into routine, national immunization programs. Future qualitative research could help deepen understanding of how individuals interpret and internalize the values of gotong royong, and clarify how these meanings influence participation in communal activities across different health programs. Expanding to other regions will help determine how widely these findings apply and inform more culturally grounded public health strategies in varied local contexts.

## 5 Conclusions

This study highlights how social norms and community engagement influence COVID-19 vaccination uptake in Indonesia, particularly through the collectivist tradition of gotong royong. The findings emphasize the need for localized public health strategies that align with distinct urban and rural social structures. These could leverage peer-driven norms in rural areas and institutional trust in urban settings. Addressing misinformation and politicization through culturally tailored communication strategies, such as community-driven messaging and digital outreach, can enhance vaccine confidence. While the findings are robust within Yogyakarta, future research should explore longitudinal and qualitative approaches to assess how collective social practices can be integrated into broader health interventions. Beyond vaccination, this study underscores the potential of community-based approaches in strengthening public health resilience and health equity in post-pandemic recovery efforts.

## Ethical approval

This study received ethical approval from the Medical and Health Research Ethics Committee Faculty of Medicine, Public Health, and Nursing at Universitas Gadjah Mada (Reference No: KE/FK/1405/EC/2022). The study protocol was also submitted to the University of Michigan Health Sciences and Behavioral Sciences Institutional Review Board (HUM00232286), which gave the study an exempt designation. Prior to any data collection procedures, all participants provided written informed consent.

## Funding

This research was supported by multiple funding sources. Abram L. Wagner was funded through the National Institute of Allergy and Infectious Diseases of the National Institutes of Health (Award Number K01AI137123). The content is solely the responsibility of the authors and does not necessarily represent the official views of the National Institutes of Health. Additional support was provided by the Office of Global Public Health and the Department of Epidemiology at the University of Michigan School of Public Health. This research was funded through the Partnerships for Enhanced Engagement in Research (PEER) program, sponsored by the United States Agency for International Development. Funders had no role in the design of the study or the decision for study findings to be published.

## Conflict of interest

The authors declare that they have no known competing financial interests or personal relationships that could have appeared to influence the work reported in this paper.
